# Metamaterial emitter for thermophotovoltaics stable up to 1400 °C

**DOI:** 10.1038/s41598-019-43640-6

**Published:** 2019-05-10

**Authors:** Manohar Chirumamilla, Gnanavel Vaidhyanathan Krishnamurthy, Katrin Knopp, Tobias Krekeler, Matthias Graf, Dirk Jalas, Martin Ritter, Michael Störmer, Alexander Yu Petrov, Manfred Eich

**Affiliations:** 1 Institute of Optical and Electronic Materials, Hamburg University of Technology, Eissendorfer Strasse 38, Hamburg, 21073 Germany; 2 Institute of Materials Research, Helmholtz-Zentrum Geesthacht Centre for Materials and Coastal Research, Max-Planck-Strasse 1, Geesthacht, 21502 Germany; 3 Electron Microscopy Unit, Hamburg University of Technology, Eissendorfer Strasse 42, Hamburg, 21073 Germany; 4 ITMO University, 49 Kronverkskii Avenue, Saint Petersburg, 197101 Russia

**Keywords:** Metamaterials, Metamaterials

## Abstract

High temperature stable selective emitters can significantly increase efficiency and radiative power in thermophotovoltaic (TPV) systems. However, optical properties of structured emitters reported so far degrade at temperatures approaching 1200 °C due to various degradation mechanisms. We have realized a 1D structured emitter based on a sputtered W-HfO_2_ layered metamaterial and demonstrated desired band edge spectral properties at 1400 °C. To the best of our knowledge the temperature of 1400 °C is the highest reported for a structured emitter, so far. The spatial confinement and absence of edges stabilizes the W-HfO_2_ multilayer system to temperatures unprecedented for other nanoscaled W-structures. Only when this confinement is broken W starts to show the well-known self-diffusion behavior transforming to spherical shaped W-islands. We further show that the oxidation of W by atmospheric oxygen could be prevented by reducing the vacuum pressure below 10^−5^ mbar. When oxidation is mitigated we observe that the 20 nm spatially confined W films survive temperatures up to 1400 °C. The demonstrated thermal stability is limited by grain growth in HfO_2_, which leads to a rupture of the W-layers, thus, to a degradation of the multilayer system at 1450 °C.

## Introduction

High-temperature emitters play a crucial role in thermophotovoltaic (TPV) energy conversion^1–9^. According to the Stefan-Boltzmann law^10^, the radiative power of a blackbody is proportional to *T*^4^. Thus, a high operating temperature is beneficial to achieve a high radiative power. At the same time, the peak of the black body spectral power density moves to shorter wavelengths with higher temperature. Thus, for the given spectral emissivity of the emitter and for a fixed bandgap position of the PV cell the conversion efficiency also grows with temperature^5^. Thermal radiation from a blackbody spans over a broad wavelength range, where most of the energy is radiated in the long wavelength region. As the photon energy is below the bandgap, the PV cell would not convert the long-wavelength photons into electricity. Additionally, since these photons eventually are absorbed nonetheless, e.g. in the housing or package, this absorbed power from low energy photons will lead to a significant increase in the PV cell temperature and thus decrease its external quantum efficiency. Front surface filters can be used to recycle the long-wavelength photons, i.e., inhibit the transmission of the low energy photons and revert them back to the emitter, which will reduce radiative losses^11–13^. Alternatively the TPV conversion efficiency can be increased if the emission at longer wavelengths is suppressed, since energy otherwise emitted e.g. by a blackbody now stays inside the emitter. In this context, spectrally selective emitters are particularly important for generating short wavelength thermal radiation. An ideal TPV thermal emitter would provide narrowband radiation with an energy just above the bandgap of the PV cell as also photon energies much higher than the bandgap pose the problem of phononic carrier thermalization with eventually and, unnecessarily, heating of the PV cell. In practical TPV systems a selective emitter which provides a step function in its spectral response, with the step positioned at the bandgap energy (*E*_*g*_) of the PV cell, is sufficient as the population of photon quantum states follows the Bose-Einstein distribution which already tails-off rapidly towards high energies. Therefore, a selective thermal emitter providing an emissivity *ε* = 1 for *E* > *E*_*g*_ and *ε* = 0 for *E* < *E*_*g*_, where *E* is the thermal photon energy, is desired. TPV research has gained a strong interest in recent years due to the advancements of structured selective emitters based on refractory materials^14^.

The selective emitters from non-refractory materials with a melting temperature below 1800 °C like Si or Pt, are usually limited to temperatures below 1000 °C^15–17^. To increase the thermal stability, refractory metals like W and Mo are beneficial^14,18^. Such refractory metals need to be structured to provide spectral selectivity^19–22^. There are two major mechanisms that lead to degradation of these structures: surface diffusion^23–26^ and oxidation^22,27–30^. The 2D and 3D structured materials contain edges on the nano scale which are subject to strong surface diffusion at temperatures below 1200 °C^25,26,31–33^. As a criterion for the emitter stability we define the preservation of its spectral characteristics. Some structural features of HfO_2_-conformal protected 3D photonic crystals (PhCs) from W, as discussed by Arpin *et al*.^2^, were retained up to 1400 °C. However, a significant increase in long wavelength absorptivity/emissivity was observed already after annealing for 1 h at 1400 °C, making the desired emission characteristic completely vanish. These changes indicate that relevant structural details were altered due to grain growth of W. The surface diffusion rate is proportional to the gradient of the edge curvature^26^. Thus, the detrimental surface diffusion effects at high temperatures can be mitigated if edge-less thin film structures are used. These are 1D layered metamaterials and Fabry-Perot resonators^22,34–36^, employing metallic and oxidic thin-films. Thin metal films are required to provide partial transparency. So far, due to oxidation, such thin metallic films could be operated up to 1000 °C, only^22^.

The focus of the present work is to investigate the thermal stability limit of such thin flat refractory W and HfO_2_ based layered metamaterial structures which avoid surface diffusion. These thin films were investigated under medium and high vacuum conditions^37^, in our case 10^−2^ to 10^−3^ and 10^−5^ to 10^−6^ mbar vacuum pressures, respectively; the latter is needed in order to suppress the oxidation of W metal. This metamaterial structure is designed to operate as the thermal emitter in a TPV system using a GaSb PV cell with a bandgap of *E*_*g*_ = 0.72 eV (≙ 1.72 μm)^38^. The presented metamaterial emitter shows unprecedented thermal stability up to the annealing temperatures of 1400 °C for 6 h duration under the vacuum of 3 × 10^−5^ mbar pressure. To the best of our knowledge, this is the highest temperature reported in the literature to date for a structured emitter. Stability in this context means that not only the structural integrity but also the spectral features, i.e. the suppression of long wavelength photon emission, are stable. Such metamaterial emitters can be utilized as thermal emitters in TPV systems. Astonishingly, these thin film structures demonstrate higher thermal stability as much coarser 2D and 3D PhCs^4,23,31^.

## Results

**Figure 1:**
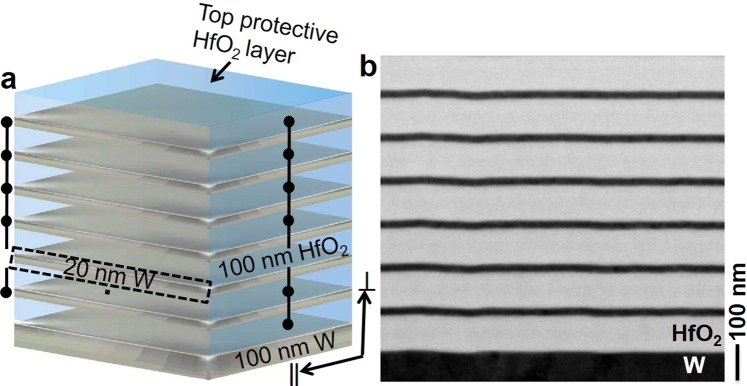
Designed and experimentally achieved 1D layered W-HfO_2_ metamaterial emitter structure. (**a**) Schematic presentation of the designed emitter structure. (**b**) STEM image shows the cross-sectional view of the as-fabricated 1D emitter structure. The Al_2_O_3_ substrate is not shown in the schematic and STEM image.

**Figure 2:**
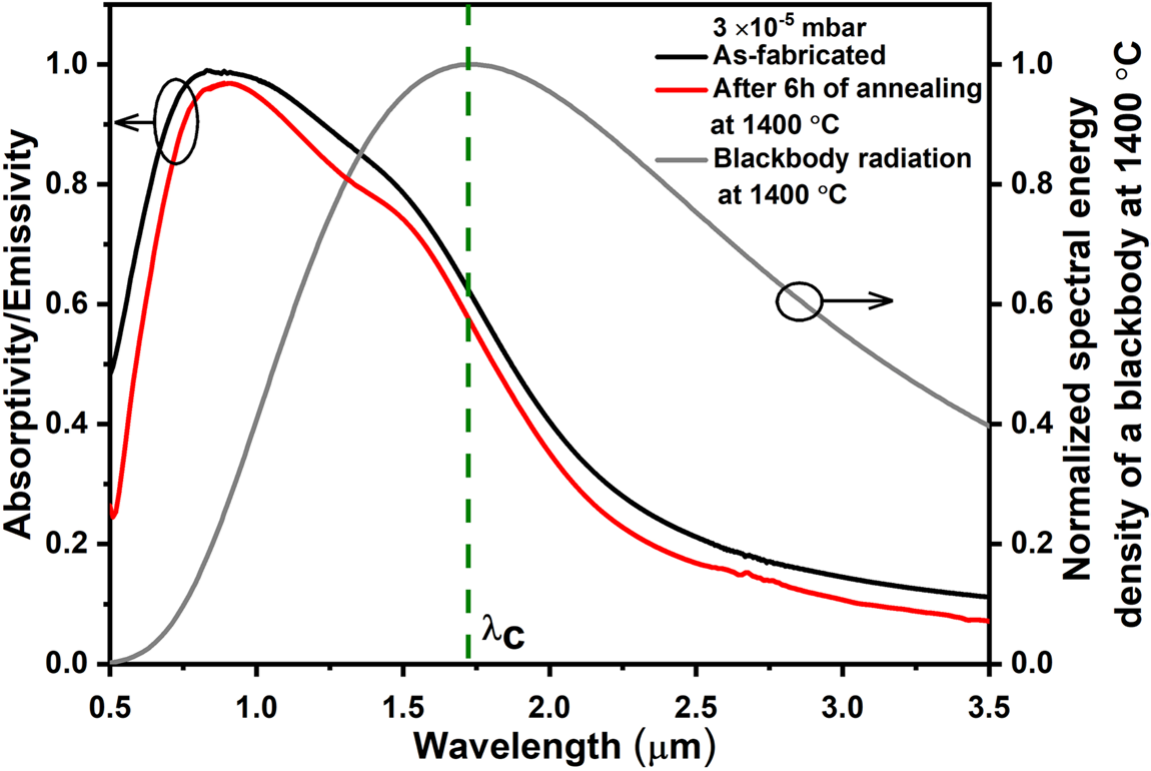
Spectral selectivity of the emitter at room temperature after annealing at high temperature. Spectral absorptivity/emissivity of the emitter measured at room temperature before and after annealing at 1400 °C for 6 h under 3 × 10^−5^ mbar vacuum pressure. λ_C_ represents the cut-off wavelength at 1.72 μm, corresponding to the bandgap of the GaSb PV cell at 0.72 eV.

A schematic of the W and HfO_2_-based layered metamaterial emitter is shown in Fig. 1a. Six bilayers of W and HfO_2_, with thicknesses of 20 and 100 nm, respectively, are sandwiched between a top protective HfO_2_ layer and bottom thick W layer, each 100 nm thick. Cross-sectional view of the high-angle annular dark-field (HAADF) scanning transmission electron microscopy (STEM) image of the as-fabricated emitter structure is shown in Fig. 1b. The number of bilayers was increased to six in comparison to four layers in our previous work^22^. This was done to avoid residual transmission through the metamaterial. According to Kirchhoff’s law of thermal radiation^39,40^ the emissivity of a hot radiating body equals its absorptivity. Therefore, we can assess the TPV-relevant spectral emissivity by measuring the absorptivity of our metamaterial layer (shown in Fig. 2). At room temperature, the as-fabricated emitter structure shows a step function-like steep spectral cutoff around 1.7 μm and low absorptivities/emissivities above the wavelength corresponding to the bandgap of the PV cell, i.e. low emission of such photons. The metamaterial emitter structure after annealing at 1400 °C for 6 h, measured at room temperature, shows similar band-edge characteristics with even a slight improvement of the spectral characteristics, e.g. a reduction of the absorptivity/emissivity at long wavelengths (Fig. 2, red trace), which is attributed to a reduced electron collision frequency due to grain growth in the tungsten layer leading to an improved metallic reflection. This effect will be discussed later.

We like to point out that our designed layered metamaterial emitter assumes a total absorptivity/emissivity of 100% around 1 µm wavelength equal to that of a blackbody (Fig. 2, black trace). At 1.7 µm wavelengths, the absorptivity/emissivity decreases rapidly due to the topological transition from an ellipsoidal to a hyperboloidal equifrequency surface in *k*-space which goes along with an increase in reflection as discussed by the authors in ref.^22^. The spectral energy density of thermal radiation emitted to the far field from the heated layered metamaterial, is certainly limited at any wavelength by the spectral energy density emitted from a blackbody at the same temperature. Therefore, for our layered metamaterial we expect the emission spectrum to be curbed towards short wavelengths by the respective blackbody characteristics which is governed by the Bose-Einstein distribution in this regime at 1400 °C. Thus, the decline in the absorptivity/emissivity of the metamaterial emitter towards short wavelengths is of no relevance here. On the other hand, towards long wavelengths, the expected metamaterial spectral emissivity is curbed by its steeply declining emissivity function which can be seen from the characteristics of the total absorptivity/emissivity. In summary, our designed layered metamaterial emitter exactly shows the desired behavior: It emits like a blackbody at short wavelengths and suppresses the emission of low energy photons above 1.7 µm wavelength (0.72 eV).

**Figure 3:**
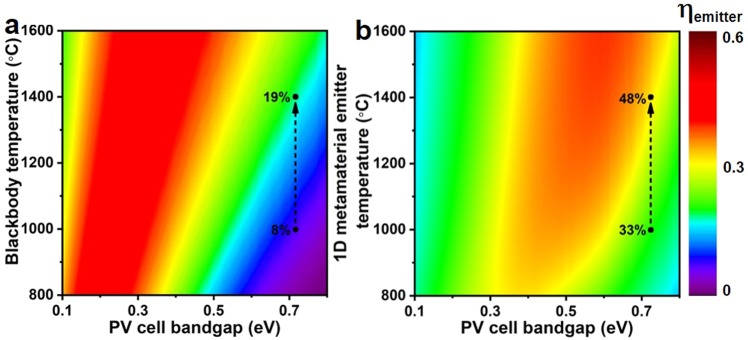
Calculated emitter efficiency of the blackbody and layered metamaterial structure. (**a**,**b**) Contour maps show the emitter efficiency *η*_*emitter*_ of a TPV cell for both blackbody and layered metamaterial emitter structure, respectively, for temperature versus PV cell bandgap energy.

In order to compare the effects of the modified emission characteristics of our layered metamaterial emitter with that of a blackbody at the same temperature, we calculated the emitter efficiencies *η*_*emitter*_ for both as a function of the temperature *T* and PV cell bandgap energy *E*_*g*_ (Fig. 3a,b). *η*_*emitter*_ is defined as the ratio of the thermal radiation energy convertible in the PV cell to the total thermal energy radiated by the emitter, as follows^41^

1 $${\eta }_{emitter}={\int }_{{E}_{g}}^{\infty }\,\frac{{E}_{g}}{E}\,\varepsilon (E){I}_{BB}(E,{T}_{emitter})dE/{\int }_{0}^{\infty }\,\varepsilon (E)\,{I}_{BB}(E,{T}_{emitter})dE$$

where *ε*, *E*, and *I*_*BB*_ correspond to the spectral emissivity, photon energy and blackbody spectral power density at the emitter temperature, respectively. At 1000 °C, the blackbody efficiency is 8% at a PV cell bandgap of 0.72 eV, which is raised to 19% when the blackbody temperature is increased to 1400 °C. In comparison to the blackbody, at 1400 °C and for the same PV cell bandgap of 0.72 eV, the calculated efficiency of our metamaterial emitter approaches 50%, which is 2.5× higher. This remarkable increase in TPV efficiency is a consequence of the sharp decline of the emitter emissivity at photon energies lower than the bandgap of the PV cell (Fig. 2). It is clear from the Stefan-Boltzmann law^10^ that the *T*^4^ characteristics of the total emitted power makes any increase of the emitter temperature highly desirable. As any structure eventually will fail at some temperature it is therefore of great importance to study in detail the mechanisms of degradation which will set temperature limits for layered metamaterial emitters in order to develop strategies for further improvements in high temperature stability.

**Figure 4:**
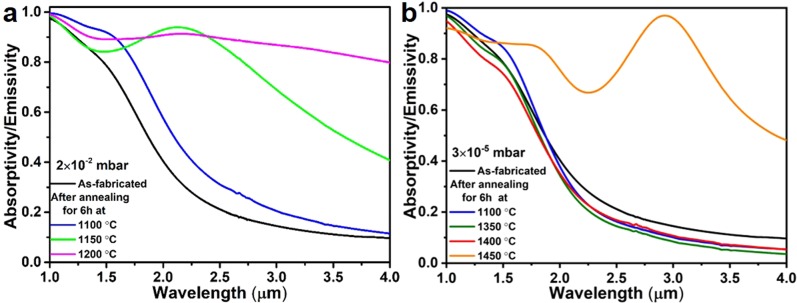
Experimental spectral absorptivity/emissivity of the layered metamaterial emitter structures. Comparison of structures as-fabricated and after 6 h annealing at various temperatures and in medium 2 × 10^−2^ mbar (**a**) and high 3 × 10^−5^ mbar (**b**) vacuum conditions.

We have therefore investigated the thermal stability of the layered metamaterial emitter structure by annealing at various temperatures (ranging from 1100 to 1500 °C) and different vacuum conditions (2 × 10^−2^, 2 × 10^−3^, 3 × 10^−5^ and 2 × 10^−6^ mbar). Different emitter structures, fabricated in the same batch with uniform spectral properties (measured at room temperature) were used for thermal annealing experiments. The duration of the annealing time was fixed to 6 h for all the structures investigated in the present work. Figure 4a shows the spectral absorptivity/emissivity of the emitter structure after annealing at 1100 °C under 2 × 10^−2^ mbar vacuum pressure. At this medium vacuum condition with a significant O_2_ partial pressure an irreversible change in the spectral selectivity (red shift, Fig. 4a-blue trace) of the emitter compared to the as-fabricated structure is observed, followed by a complete degradation of the spectral features at 1150 and 1200 °C. These changes are accompanied by a strong increase of the emission of long wavelength photons, typical for an arbitrary grey body. Thus, a detrimental change of the band-edge characteristics of the emitter is observed.

**Figure 5:**
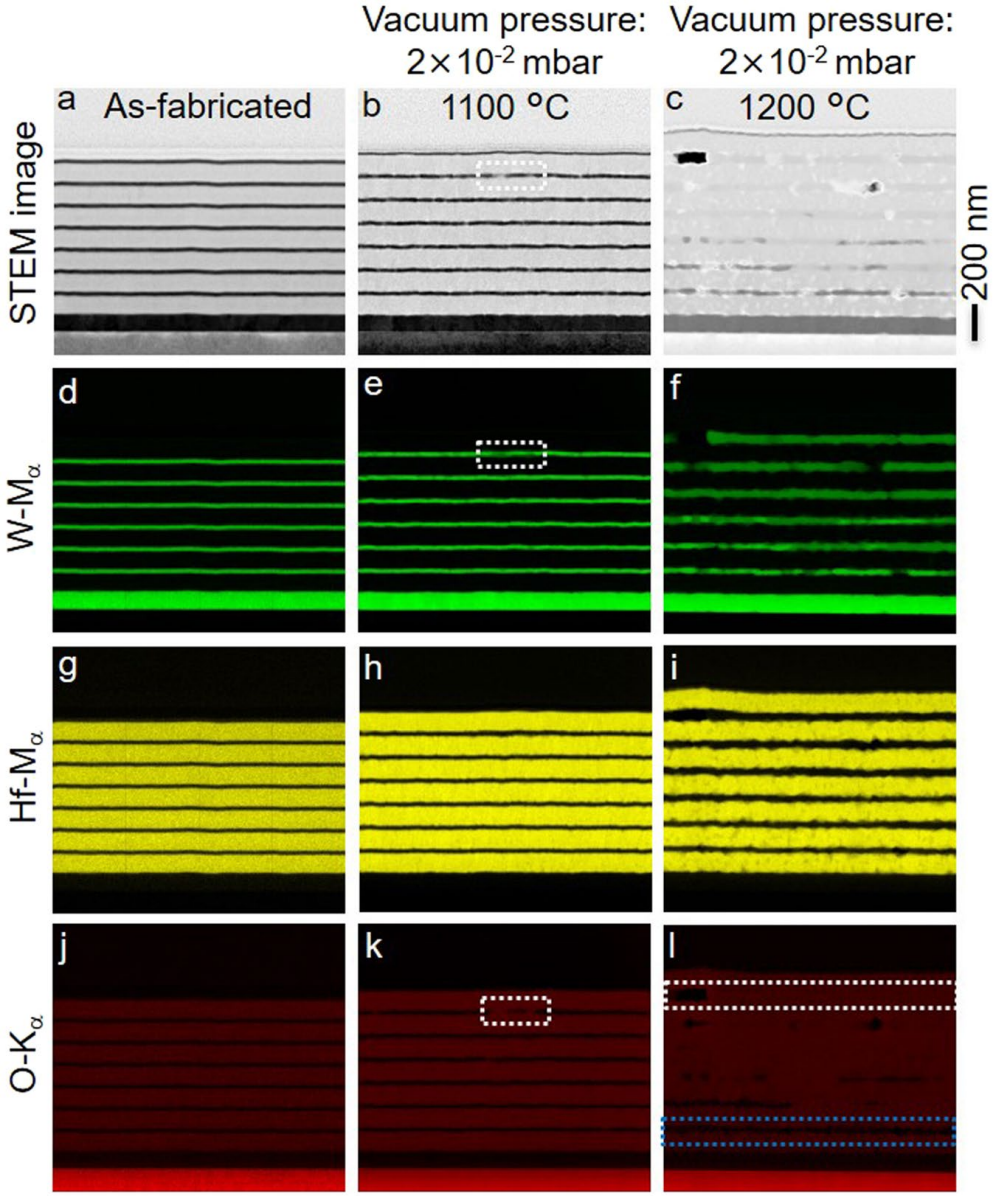
Morphology and degradation of the layered metamaterial emitter due to oxidation at a medium vacuum condition of 2 × 10^−2^ mbar. STEM image (**a**–**c**) and element mapping (**d**–**l**, spectrum images for W, Hf and O) of the emitter structure, for as-fabricated and annealed for 6 h at 1100 and 1200 °C. Note that a gold cover layer is deposited prior to the FIB milling to circumvent charging of the substrate.

To identify the underlying physics of the changes in spectral characteristics, STEM, energy-dispersive x-ray spectroscopy (EDS) and X-ray diffraction (XRD) analyses were performed on the as-fabricated and annealed structures. In the case of the as-fabricated structure, the STEM image (Fig. 5a) clearly shows the stack of W and HfO_2_ layers on an Al_2_O_3_ substrate with smooth interfaces between the adjacent layers. For the structure annealed for 6 h at 1100 °C and 2 × 10^−2^ mbar vacuum pressure (Fig. 5b) contrast changes in the top W film at certain regions are observed, and the corresponding element mappings for W, Hf and O analyses are presented in Fig. 5d–l in the respective vertical columns. Both, the STEM image and element mapping of W show greyish and dark green shades (highlighted by the white-dotted rectangles), respectively, in the degraded region of the top W film. Most importantly, the O mapping shows the existence of O in this region of the top W film. Also, the W film roughness increases, while, a grainy network can be seen in the STEM and element maps.

Annealing the layered metamaterial emitters at temperatures 1100 °C and above at 2 × 10^−2^ mbar medium vacuum condition leads to oxidation of the W layers in the emitter structure. In particular, when the emitter is annealed at 1200 °C (see Fig. 5, right column), the top W layers are more strongly degraded than the bottom W layers, while the bottom 100 nm thick W layer is left unchanged. The element mapping of W and O (Fig. 5f,l) shows the presence of O in the degraded regions of the W films. An O concentration gradient is clearly seen in the layered structure, where, after annealing, the relative percentage of the O content is high in the top W layers, and gradually decreasing towards the bottom W layers (also see Supplementary Fig. 1). Thermal annealing at 1200 °C under medium vacuum conditions, i.e. 2 × 10^−2^ mbar, two tungsten oxides, WO_2_ and WO_2.9_, are considered to be formed in the emitter structure. The XRD pattern of the metamaterial emitter annealed at 1200 °C (see Supplementary Fig. 2) confirms the formation of the monoclinic WO_2_ phase by proof of several diffraction peaks at 2θ = 25.8°, 36.8° and 59.7° corresponding to (011), (200) and (031) planes, respectively. Diffraction peaks for WO_2.9_ are not observed in the XRD patterns since the WO_2.9_ is sublimated and only WO_2_ is left in the emitter structure. The sublimation of the WO_2.9_ is observed at 1200 °C, whereas WO_2_ sublimates at 1500 °C (Supplementary Fig. 3). The sublimated WO_2.9_ leaves voids in the W layer behind (Fig. 5c,f) and is deposited as a blue coating^42–46^ onto the radiation shield of the heating chamber (see Supplementary Fig. 3b).

We have to consider two different mechanisms; both can lead to the oxidation of W layers: inter-layer diffusion of O from HfO_2_ to W, and O_2_ diffusion from the external environment at medium vacuum conditions and elevated temperatures. Refractory W and HfO_2_ are chosen in the present study due to their high-melting points^41^. Furthermore, the free enthalpy for the formation of HfO_2_ is −909 kJ/mol, which is much lower than the free enthalpy of formation of WO_2_ (−530 kJ/mol)^47,48^ which gives a thermodynamic explanation for the higher stability of HfO_2_. In other words, a reduction of HfO_2_ leading to a W oxidation would lead to a Gibbs free enthalpy of +379 kJ/mol, i.e. a highly endergonic reaction. Consequently, a W oxidation at the cost of HfO_2_ reduction can be neglected even at highly elevated temperatures (see SI for further explanation). Thus, a reaction of the W layers with the residual ambient O_2_ represents the main oxidation mechanism. This interpretation is supported by oxygen gradient observed after 6 h of annealing showing that the topmost thin W layer is completely oxidized whereas the bottom thin W layer is unaffected. Oxygen content is quantified using the O-K line intensity in STEM image (Fig. 5l) and Supplementary Fig. 1b, where 100% O is observed in the top thin W layer, white-dotted rectangle in Fig. 5l, and 0% O is observed in the bottom thin W layer, blue-dotted rectangle in Fig. 5l. The thin W layers between top and bottom W layers show an O gradient, where the O content is decreasing from 100% to 0% in the direction from top to bottom layers. In the case of the metamaterial emitter annealed at 1100 °C, within a period of 6 h, O_2_ transport from the external environment into the emitter structure is insufficient to oxidize the entire topmost W film. It turned out, that due to temperature dependent diffusion of O_2_, a complete replenishment of oxygen in the top W film and concentration gradient in the subsequent films is observed at 1200 °C (Supplementary Fig. 1b). No pinholes were observed in HfO_2_ layers (STEM image, Fig. 5h,i), thus, O diffusion in HfO_2_ at 2 × 10^−2^ mbar vacuum pressure is attributed to grain boundary diffusion and lattice vacancies. The formation of tungsten oxides in the emitter structure after annealing at medium vacuum condition of 2 × 10^−2^ mbar can be summarized as: 1. O_2_ diffusion from the outer atmosphere to the top W layer through the HfO_2_ layers. 2. Reaction of the upper W layers with O_2_ and formation of monoclinic WO_2_ and volatile WO_2.9_. 3. Eventually, oxidation of the deeper W layers and subsequent sublimation of the formed WO_2.9_ will continue in a sequential manner with progressing annealing time. Thus, O_2_ diffusion from the external environment is the limiting factor in thermal stability of a W/HfO_2_ layered metamaterial emitter at medium vacuum conditions.

**Figure 6:**
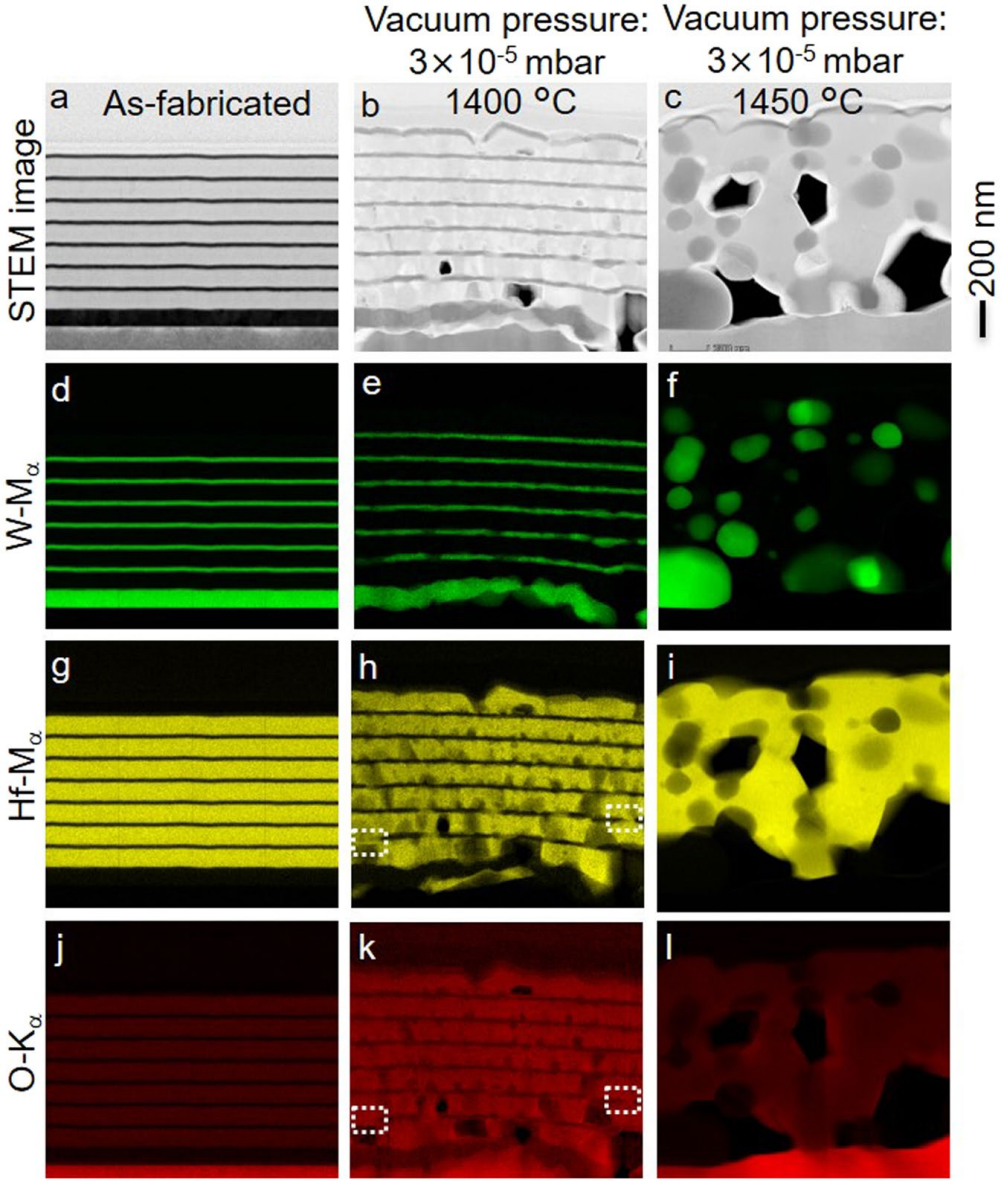
Morphology and degradation of the layered metamaterial emitter due to grain growth in the HfO_2_ at an annealing temperature of 1400 °C and 1450 °C at a high vacuum condition of 3 × 10^−5^ mbar. STEM image (**a**–**c**) and element mapping (**d**–**l**, spectrum imaging for W, Hf and O) of the emitter structure, for as fabricated and annealed for 6 h at 1400 and 1450 °C.

By reducing the vacuum pressure to 2 × 10^−3^ mbar, the optical absorptivity/emissivity of the emitter shows a slightly enhanced thermal stability up to 1100 °C and oxygen content is not observed in the W layers (Supplementary Figs 4a and 5-right column). Spectral broadening is observed after annealing at 1200 °C and above, presumably due to the discussed oxidation of the W layers which, for a fixed temperature, shows a decreased rate due to the O_2_ partial pressure (assumed to be 21% of vacuum pressure)^49^ reduced by one order of magnitude. Consequently, we further reduced the vacuum pressure by another two orders of magnitude to 3 × 10^−5^ mbar and the thermal stability of the metamaterial emitter was investigated. Figure 4b shows the spectral absorptivity/emissivity of the emitter structures after annealing for 6 h at 1100, 1350, 1400 and 1450 °C. We find that the spectral selectivity, expressed as the decrease of the absorptivity/emissivity above 1.7 µm wavelength, is retained up to 1400 °C. The plasmonic properties of W are even slightly improved after the thermal annealing process, which is expressed as a sharper spectral transition and lower thermal emission in the near-infrared region of the annealed metamaterial compared to the as-fabricated. The small grains or high proportion of grain boundaries in the W layers of the as-fabricated metamaterial lead to an increase of the effective collision frequency of the electrons in W. This is associated with a larger imaginary part of the W dielectric constant, i.e. the loss term in the Drude model, and a broadening of the spectral response. An increased grain size (Fig. 6a,b) is observed after thermal annealing. This reduction in volume fraction of grain boundaries in the W layers reduces the electron collision frequency. Thus, after annealing, the metallic character of the W layers is improved and sharper transitions, and lower absorptivities (and, consequently, lower emissivities) due to better metallic reflection above the cut-off wavelength is also observed (see Figs 2 and 4b, red traces). We conclude that, at vacuum pressures of 3 × 10^−5^ mbar and for annealing times of 6 h, oxidation plays no role as degradation mechanism of the W/HfO_2_ layered metamaterial.

After thermal annealing at 1400 °C, the cross-sectional STEM image shows microstructural changes in W and HfO_2_ films, Fig. 6b. However, no significant deformation in the W films is observed, an increase of W grain size is clearly visible (Fig. 6d,e). Most importantly W is still confined in its layered geometry, so that a grain growth can only proceed within a, i.e. intra-layer. In contrast, we observe the grain growth and void formation in HfO_2_. For the as-fabricated emitter structures, the XRD pattern shown in Supplementary Fig. 2 exhibits the polycrystalline nature of monoclinic HfO_2_ with preferred grain orientation in (−111) plane at 2θ = 28.3°, whereas the other barely visible Bragg peaks at 2θ = 50.9° and 62.4°, correspond to (221) and (−312) planes, respectively. After annealing the emitter structure at 1400 °C for 6 h, the Bragg peaks of (−111) and (−312) planes at 2θ = 28.3° and 62.4°, respectively, exhibit comparable intensity level, thus some uncertainty in the grain orientations (no preferred grain orientation) is expected. Moreover, randomly oriented single crystallites of HfO_2_ with sharp grain boundaries can be clearly seen in the Supplementary Fig. 6a. Although a noticeable change in the effective film thickness of the metamaterial emitter structure is observed after annealing at 1400 °C (Fig. 6b), due to grain growth in W and HfO_2,_ and void formation in HfO_2_, we emphasize that the emitter retains its spectral selectivity even after 6 h annealing at 1400 °C. Since, the total amount of W and HfO_2_ content does not change in the structural transformation process, no drastic changes in optical spectra is observed in agreement with an effective medium approximation^22^.

Only when the temperature is further increased to 1450 °C, a drastic change in the spectral absorptivity/emissivity is observed (Fig. 4b). As shown by the STEM and element maps (Fig. 6, right column), the complete degradation of the emitter structure occurs.

### Based on the STEM analyses, we assume that degradation at high vacuum conditions proceeds in two steps

1. During annealing of the emitter structure at 1400 °C, growing HfO_2_ grains locally advance to the respective HfO_2_ layer boundary. The as-fabricated emitter structure contains small grains of HfO_2_ with an average size of 15.7 nm (Supplementary Fig. 11). During annealing, these small grains are particularly unstable, owing to their large surface-to-volume ratio, and reduce their surface energy by growing to larger grains by coalescing the adjacent small grains. Supplementary Fig. 11 shows a gradual increase in average grain size of the HfO_2_ by rising the annealing temperature from room temperature to 1450 °C. At certain regions of the W-HfO_2_ interfaces, growing HfO_2_ grains (Supplementary Fig. 6) are eventually protruding into the W layers and deforming them locally with their sharp edge-like shape.

A noticeable weakening of the layered structure integrity is observed. Magnified STEM image of the Supplementary Fig. 6b,c clearly show the HfO_2_ grain protrusions in W film. Also, the white-dotted rectangles in Fig. 6h,k depict both Hf and O content inside the deformed W regions. Moreover, voids occur in HfO_2_ owing to the grain growth at constant density. As a result, the elemental mapping of Hf (Fig. 6h) shows regions of black, dark and bright yellow regions (no, low and high signal intensities, respectively) in the HfO_2_ layers due to formed voids on top of the cross-section, voids deeper inside and pristine HfO_2_, respectively.

2. HfO_2_ grain penetration at the interface leads to local ruptures of the W thin-film. Then, well known for the behavior of structured W at high temperatures^4,23,31^, the broken W structures tend to reduce their surface energy by self-diffusion of W atoms and convert into round W particles, which are thermodynamically favoured because of their minimized surface-to-volume ratio. Finally, large voids are observed in the emitter structure due to the migration of HfO_2_ and W.

We can conclude that, as long as W is kept spatially confined in edge-less layers, this spatial confinement stabilizes the W-HfO_2_ multilayer system to temperatures unprecedented for other nanoscaled W-structures. Only when this confinement is broken (here, by protrusions from growing adjacent HfO_2_ grains) the W starts to show the well-known self-diffusion behavior transforming to spherical shaped W-islands. We point out that at a high vacuum condition of 3 × 10^−5^ mbar no detectable amount of oxygen is observed in these W-rich regions (Fig. 6l). The XRD patterns for the emitter structure annealed at 1400 and 1450 °C (see Supplementary Fig. 2) shows no additional diffraction peaks, which can be attributed to W oxides (WO_2_ and WO_2.9_). These findings confirm that, at high vacuum conditions, oxidation of W is no relevant degradation mechanism for the emitter structure. Instead, at very high temperatures, at 1450 °C, grain growth in the HfO_2_ is the limiting factor limiting the structural and hence spectral stability of our 1D layered metamaterial emitter.

**Figure 7:**
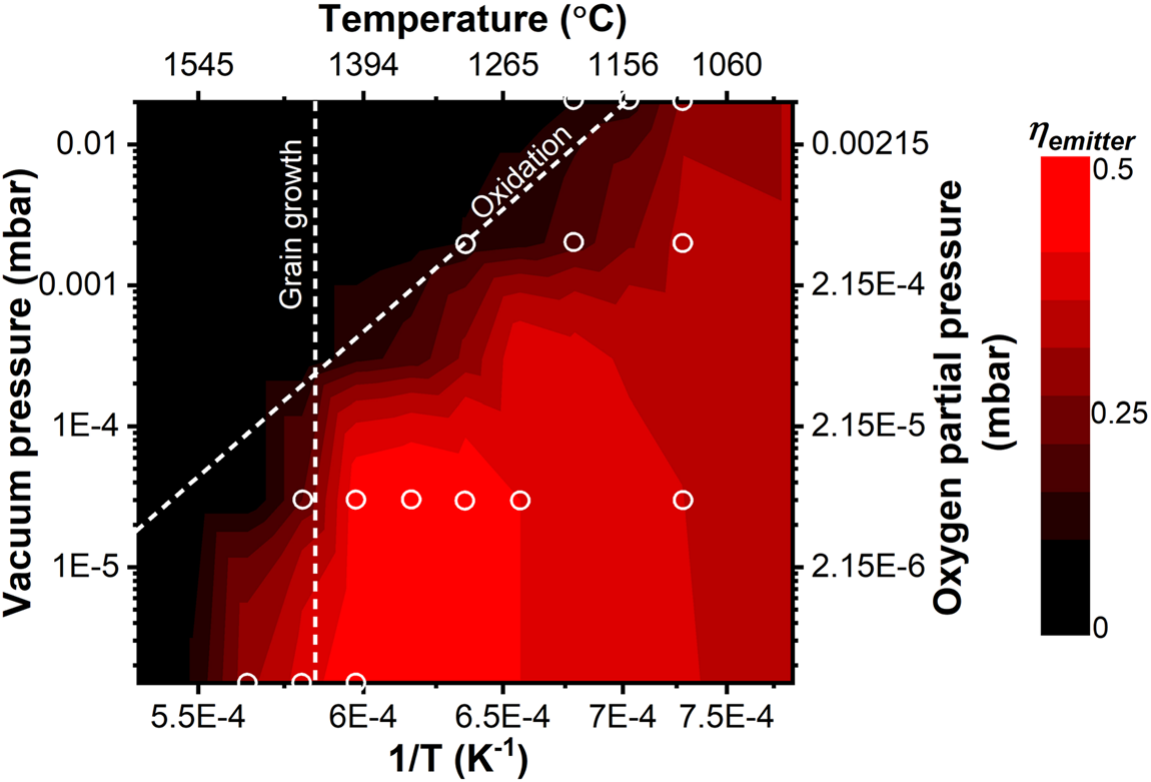
The spectral degradation of the layered metamaterial emitter encompassing oxidation at medium vacuum conditions and temperatures <1400 °C, following an Arrhenius law, and microstructural changes at high vacuum conditions and temperatures >1400 °C when oxidation is suppressed. The false-colored map shows the interpolated emitter efficiency for PV cell with bandgap at 1.7 µm after 6 h annealing at defined vacuum pressure and temperature. Emitter efficiencies corresponding to the measured structures are highlighted with open circles.

To summarize the results, Fig. 7 shows the false-colored map of the calculated emitter efficiency as a function of annealing temperature and vacuum pressure. The *η*_*emitter*_ is calculated using Eq. 1 for the experimental spectra of the emitter annealed at different vacuum conditions, i.e., 2 × 10^−2^, 2 × 10^−3^, 3 × 10^−5^ and 2 × 10^−6^ mbar, shown in Fig. 4 and Supplementary Fig. 4. The low emitter efficiency is directly related to the changes in spectral features, e.g. the disappearance of the band-edge characteristics due to the degradation of the layered metamaterial emitter. At medium vacuum conditions, oxidation plays a major role in the degradation. The amount of O_2_ diffusing through HfO_2_ in 6 h is depending on the temperature and on the O_2_ partial pressure, i.e. on vacuum pressure or atmospheric composition and follows an Arrhenius law of thermal activation. We assume that the same efficiency degradation will be achieved if the same amount of O_2_ diffuses. Thus, the activation energy for O_2_ diffusion can be derived via an Arrhenius fit. The plot of the logarithmic vacuum pressure versus 1/*T* shows a linear relationship, i.e., log(p) ∝ *E*_*a*_/*k*_*B*_*T*, where p, *E*_*a*_ and *k*_*B*_ are vacuum pressure, activation energy and Boltzmann’s constant, respectively. We fitted the slope of constant efficiency of 30% (slanted dashed line in Fig. 7) and obtained an activation energy *E*_*a*_ ~ 2.2 eV. This value shows a good agreement with the experimentally measured interstitial diffusion of O_2_ in HfO_2_^50,51^. The thermal dependence of O_2_ diffusion kinetics also explains the observations made for different temperatures. The degradation mechanism rate follows the Arrhenius dependence, which varies exponentially with (1/T) and linearly with time, where a relatively small reduction of the operating temperature will increase the durability with an order of magnitude. In our structure it is approximately 4× increase in durability for the reduction of every 100 K. Moreover, the calculated oxygen partial pressure at equilibrium for the formation of tungsten oxides at 2 × 10^−2^ mbar vacuum condition (Supplementary Fig. 10) is significantly lower than the oxygen partial pressure in our experiments. Since, we still observe intact W layers at elevated temperatures up to 1400 °C, we have to deduce on an efficient diffusion hindrance by the top-most HfO_2_ layer^50,52,53^.

At high vacuum conditions the level of constant efficiency deviates from the Arrhenius dependence. Namely, the efficiency decreases dramatically above 1450 °C independent of vacuum pressure. The vertical dashed line corresponds to the structural degradation mechanism of the emitter due to grain growth in the HfO_2_ layers, which is pressure independent and is the limiting factor at high vacuum conditions. Surprisingly, at high vacuum conditions the thermal stability of presented thin-film metamaterial is not limited by the metallic but by the ceramic constituent while thin W layers of 20 nm are likely to survive even higher temperatures.

## Discussion

In this work, we have systematically investigated the degradation mechanisms of W-HfO_2_ layered metamaterial emitter structures employing spatially confined edge-less W-nanolayers at high temperatures and various vacuum conditions. Such layered metamaterial emitter structures are spectrally selective and possess band-edge absorbing/emitting characteristics. Due to this particular spectral band-edge feature, the maximum efficiency of a TPV-system could be increased to 48% at a temperature of 1400 °C. Under vacuum pressure below 3 × 10^−5^ mbar, our layered metamaterial emitters exhibit an outstanding thermal durability up to 1400 °C over 6 h after which the spectral band-edge characteristics is still retained. To the best of our knowledge this temperature is the highest reported for selective emitters, so far.

Also, we have clarified the potential degradation mechanisms initiating the structural instability at medium and high vacuum conditions. At medium vacuum condition, i.e., 2 × 10^−2^ mbar and below the maximum temperature of 1100 °C, it is clearly observed that the residual O_2_ in the annealing chamber diffuses into the emitter structure, leading to a degradation of the spectral band-edge characteristics due to a layer-by-layer oxidation of W. At high vacuum condition of 3 × 10^−5^ mbar and temperatures at 1450 °C, the grain growth in the HfO_2_ layers causes a deformation of the W layers which finally ruptures the layered structure and consequently sets the limits for the thermal stability of the emitter. Most importantly, we find that as long as W is kept spatially confined in edge-less layers, this spatial confinement stabilizes the W-HfO_2_ multilayer system to temperatures unprecedented for other nano-scaled W-structures. Only when this confinement is broken (here, by protrusions from growing adjacent HfO_2_ grains) the W starts to show the well-known self-diffusion behavior transforming to spherical shaped W-islands. Beyond applications as TPV emitter these results are important for understanding the thermal stability of other nanostructured materials, as for example multilayer hard coating for cutting tools inserts^54,55^. Further studies should be directed towards the implementation of O_2_ diffusion barriers or inert gas conditions, which potentially can stabilize the W-HfO_2_ metamaterial up to 1400 °C even at medium vacuum conditions. Using sputtering as a low-cost, versatile and scalable fabrication method, layered metamaterial emitter structures can then play an important role in the commercialization of TPV. The thermal stability limit of the metamaterial emitter structure beyond 1400 °C can potentially be achieved by controlling the grain growth in the HfO_2_ layers, e.g., by doping. Also, other types of refractory oxidic layers, e.g. stabilized ZrO_2_, Al_2_O_3_, MgO, etc., can be investigated.

## Methods

### 1D metamaterial emitter structure fabrication

Multilayers of HfO_2_ and W films are deposited onto cleaned 5 × 5 mm^2^ single crystalline sapphire substrates ([1-102] orientation) by radio frequency and direct current magnetron sputtering at a rate of 0.2 and 0.09 nm s^−1^, respectively. All the W and HfO_2_ layers are deposited sequentially at an argon (99.99999%) gas pressure of 2 × 10^−3^ mbar. The base pressure of the sputtering chamber was below 10^−7^ mbar. The sputtering targets W (99.95%) and HfO_2_ (99.95%) were purchased from Sindlhauser Materials.

### Thermal annealing

Thermal annealing measurements were performed in a high-temperature heating stage (Linkam, TS1500) for 2 × 10^−2^ and 2 × 10^−3^ mbar pressures using a rough vacuum pump and turbomolecular pump, respectively, and in a high-temperature vacuum furnace (RD-G WEBB) for 3 × 10^−5^ and 2 × 10^−6^ mbar pressures, respectively. All the samples were annealed for 6 h at the specified temperature. The temperature was ramped at a rate of 10 °C min^−1^.

### Reflection measurements

Reflection spectra of the emitter structures before and after annealing at high-temperatures are measured using a UV-Vis-NIR spectrometer (PerkinElmer Lambda 1050) and a Fourier transform infrared spectrometer (FTIR-Vertex 70, Bruker), in the ranges of 0.3 to 2.5 μm and 2 to 10 μm, respectively. The optical absorptivity α is obtained by α = 1 *− ρ − τ*, where *ρ* and *τ* are reflectivity and transmissivity. Due to a 100 nm thick bottom W layer we realized *τ* = 0 in the measured spectral range. Thus, absorptivity, α = 1 *− ρ*, can be directly deduced from the reflection spectra.

### Morphology and elemental analysis of the emitter structure

Cross-sectional STEM samples were prepared with a focussed-ion beam (FIB, FEI Helios G3 UC) machine using a 30 keV gallium ion beam, and transferred to Cu lift-out grids via lift-out technique. To prevent charging during FIB preparation, the samples were sputtered with a 20 nm layer of Au before FIB preparation. The final thickness of the lamellae was around 100 nm. An FEI Talos F200X transmission electron microscope equipped with a high brightness Schottky-FEG (X-FEG) and a four-quadrant SDD-EDS systems (solid angle of 0.9 srad) was used for HAADF imaging and EDS analysis. HAADF images were acquired with a take-off angle of 16–82 mrad. Bright field STEM images were acquired with an objective aperture to enhance the contrast of individual grains. Spectrum images were obtained using a probe current of 1 nA and a dwell time of 5 µs per pixel. Resolution of the spectrum image is 1024 × 1024 pixels, 1.5 nm in size, resulting in a horizontal field of view of 1.56 µm. Velox 2.1 (FEI) was used for data acquisition and visualization. For SI the energies of following elements were used: Al-K_α_ (1.49 keV), O-K_α_ (0.52 keV), Hf-M_α_ (1.64 keV) and W-M_α_ (1.77 keV).

### XRD measurements

XRD measurements were conducted using a Bruker D8 advanced diffractometer. Cu Kα (λ = 0.15405 nm) radiation was used to investigate the emitter structure. The measurements were performed using parallel beam geometry. The diffraction patterns (2θ from 20° to 90°) were recorded with an increment of 0.04° and a step time of 16 s. HfO_2_ grain size was calculated using Scherrer formula^56,57^ from the (−111) reflex.

## Supplementary information

SUPPLEMENTARY INFO
