# Supplementary material for: Metamaterial emitter for thermophotovoltaics stable up to 1400 °C
Source: Sci Rep. 2019 May 10;9:7241. doi: 10.1038/s41598-019-43640-6 (PMC6510906; doi:10.1038/s41598-019-43640-6)
Supplement: Supplementary file 1 — SUPPLEMENTARY INFO [file 41598_2019_43640_MOESM1_ESM.docx]

**Supplementary Information**

Metamaterial emitter for thermophotovoltaics stable up to 1400°C

Manohar Chirumamilla^1^*, Gnanavel Vaidhyanathan^2^, Katrin Knopp^1^, Tobias Krekeler^3^, Matthias Graf^1,2^, Dirk Jalas^1^, Martin Ritter^3^, Michael Störmer^2^, Alexander Yu Petrov^1,4^, and Manfred Eich^1,2^

^1^Institute of Optical and Electronic Materials, Hamburg University of Technology, Eissendorfer Strasse 38, Hamburg 21073, Germany.

^2^Institute of Materials Research, Helmholtz-Zentrum Geesthacht Centre for Materials and Coastal Research, Max-Planck-Strasse 1, Geesthacht 21502, Germany.

^3^Electron Microscopy Unit, Hamburg University of Technology, Eissendorfer Strasse 42, Hamburg 21073, Germany.

^4^ITMO University, 49 Kronverskii Avenue, Saint Petersburg 197101, Russia.


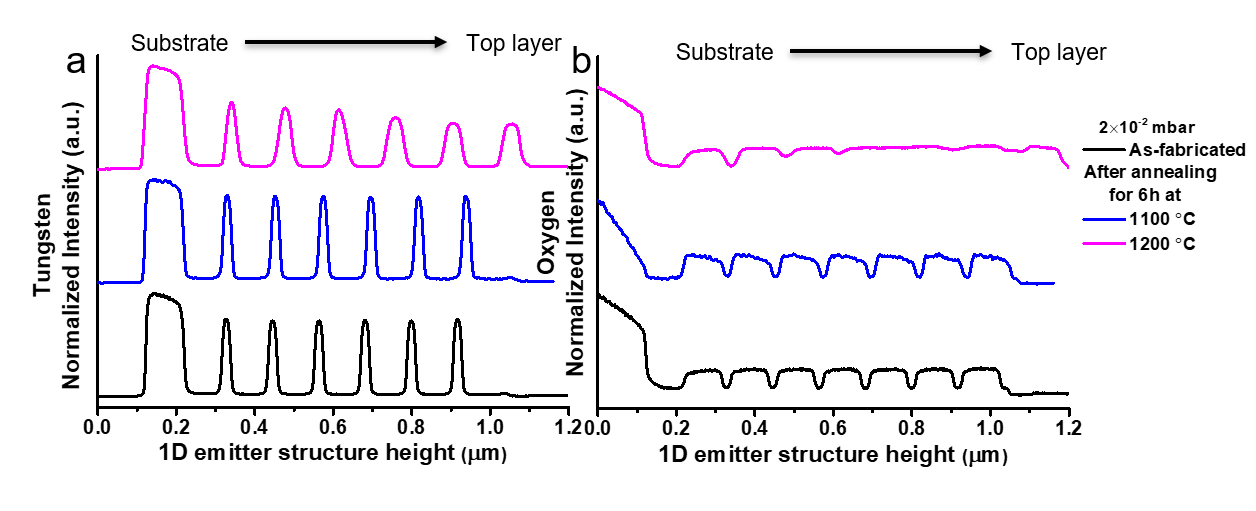


**Supplementary Figure 1 | Elemental analysis**. EDS linescans of (**a**) W (W-L line) and (**b**) O (O-K line) along the cross-section of the substrate, from sapphire substrate to the top layer of the emitter structure, as fabricated (black line) and annealed at 1100 °C (blue line) and 1200 °C (magenta line) under 2×10^-2^ mbar vacuum pressure.


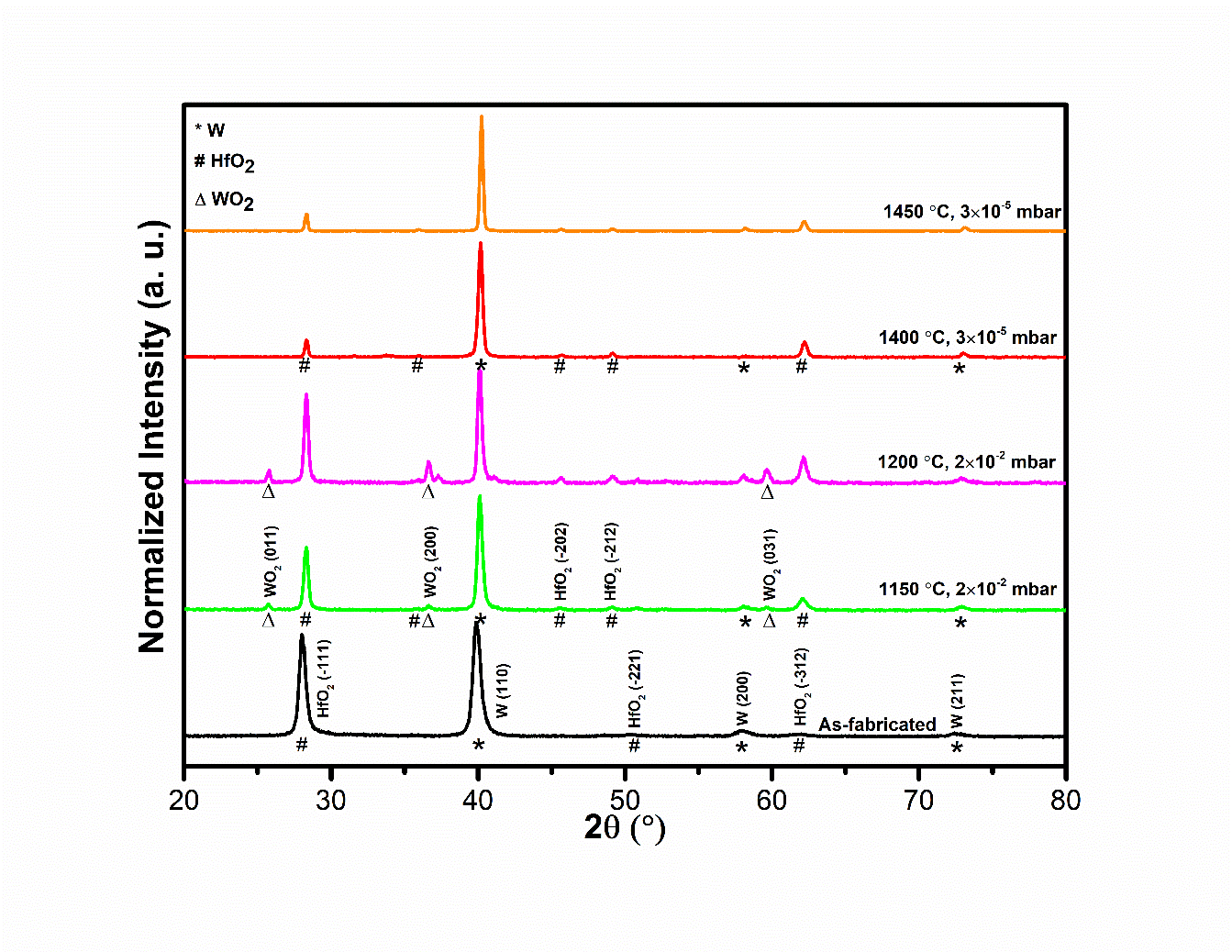


**Supplementary Figure 2 | XRD analysis of the emitter annealed at high and low vacuum pressures**. XRD patterns of the emitter structure for as-fabricated and annealed according to labelling, which were normalized with respect to dominant (110) peak of bcc W.

The XRD pattern of the as-fabricated structure reveals the polycrystalline structure of the sputtered W and HfO_2_ films, where W exhibits the bcc structure (JCPDS 00-004-0806) with α-phase, (110), (200) and (211) planes at 2θ = 40.3°, 58.3° and 73.2°, respectively, and HfO_2_ has a monoclinic structure (JCPDS 034-0104), (‑111), (221) and (-312) planes at 2θ = 28.3°, 50.9° and 62.4°, respectively. When the annealing temperature is raised to 1100 °C the diffractogram remains virtually unchanged (not shown). Since the percentage of WO_2_ in the top W layer is very low (Figure 5b), subtle structural modifications are not detected in the XRD pattern. When the annealing temperature is raised to 1150 °C, in addition to the W and HfO_2_ diffraction peaks observed in the as-fabricated case, additional peaks (at 2θ = 25.8°, 36.8° and 59.7°) are observed which are ascribed to the WO_2_ (JCPDS 32-1393) at (011), (200) and (031) planes, respectively. The new phase is caused by diffusion of O_2_ from the external environment during annealing. Narrowing of the diffraction peaks at 2θ = 28.3° and 40.3° after annealing at high temperatures, corresponding to the HfO_2_ and W at (‑111) and (110) planes, respectively, confirms the grain size increment in W and HfO_2_.


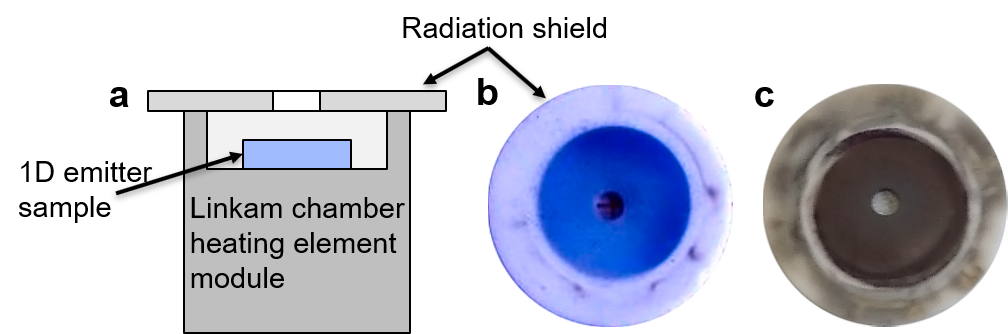


**Supplementary Figure 3 | Formation of WO_2.9_ and WO_2_ by sublimation at high temperatures and medium vacuum conditions. a**, Schematic of the heating element module in the Linkam vacuum chamber. **b** and **c**, Sublimation of WO_2.9_ and WO_2_ deposited on the radiation shield after annealing the emitter structure at 1100 °C for 6 h under 2×10^-2^ mbar, and 1500 °C under 2×10^-3^ mbar, respectively.


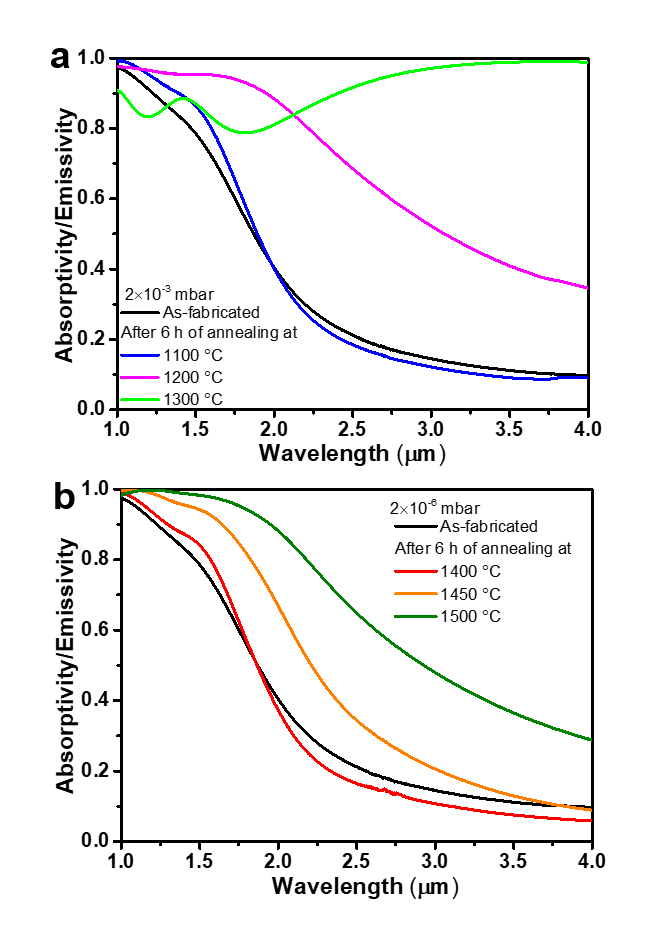


**Supplementary Figure 4 | Spectral stability and the band-edge characteristics at 2×10^-3^ and 2×10^-6^ mbar vacuum conditions**. **a** and **b**, Spectral absorptivity/emissivity of the emitter structure for as-fabricated samples and samples after annealing for 6 h at various temperatures.

Supplementary Figure 4a shows the absorptivity/emissivity spectra of the layered metamaterial emitter for as-fabricated and annealed at 1100, 1200 and 1300 °C under 2×10^-3^ mbar vacuum pressure. For the emitter structure annealed at 1100 °C, the spectral absorptivities/emissivities remains unchanged. As seen in STEM and element mappings, Supplementary Figure 5, WO_2_ is not observed in W layers, which emphasizes the critical importance of the O_2_ free environment in the annealing chamber. Spectral degradation at 1200 and 1300 °C is due to the formation of WO_2_, Supplementary Figure 7.

Further, the thermal stability of the layered metamaterial emitter was investigated by annealing for 6 h at 2×10^-6^ mbar. The spectral response of the emitter for as-fabricated and annealed at 1400, 1450 and 1500 °C is shown in Supplementary Figure 4b and comparable to the studies performed at 3×10^-5^ mbar vacuum pressure (Figure 4b in the main text). Excellent spectral/structural stability is observed until 1400 °C (Supplementary Figures 4b, 8 and 9).


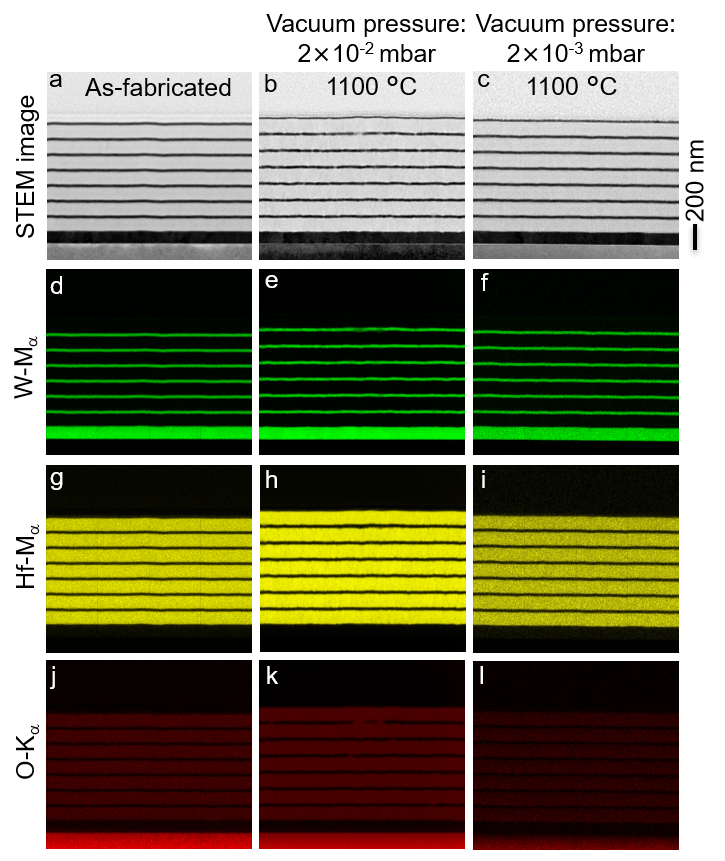


**Supplementary Figure 5 | Oxidation of top W layer after annealing the emitter at 1100 °C under 2**×**10^-2^ mbar vacuum pressure**. Emitter structure annealed at 1100 °C shows deformation in the top W layer at 2×10^-2^ mbar pressure, whereas structural stability at 2×10^-3^ mbar pressure is observed.


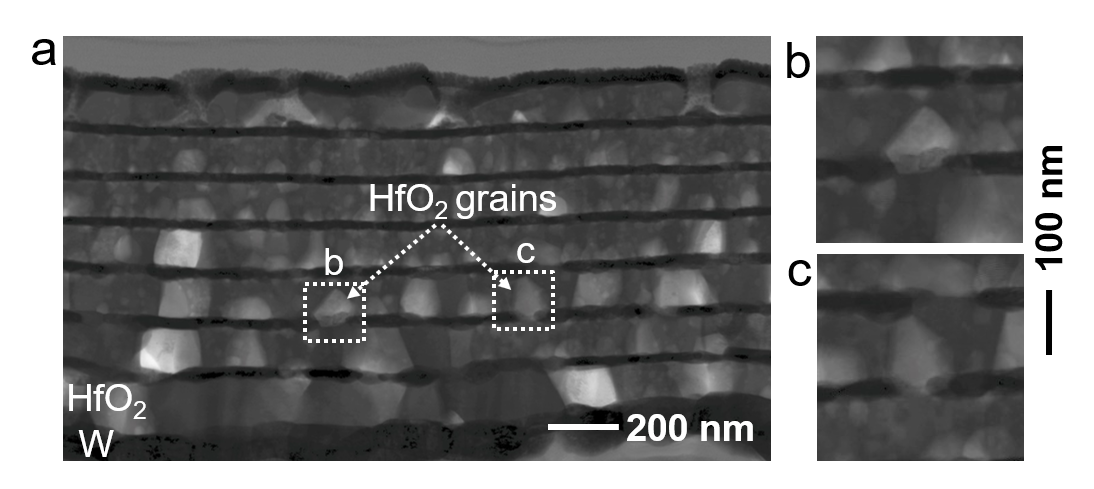


**Supplementary Figure 6 | a**, Grain growth in the HfO_2_. Bright field STEM image of the emitter structures annealed at 1400 °C under 3×10^-5^ mbar pressure for 6 h. **b** and **c**, Magnified images of the HfO_2_ protrusions in W film.


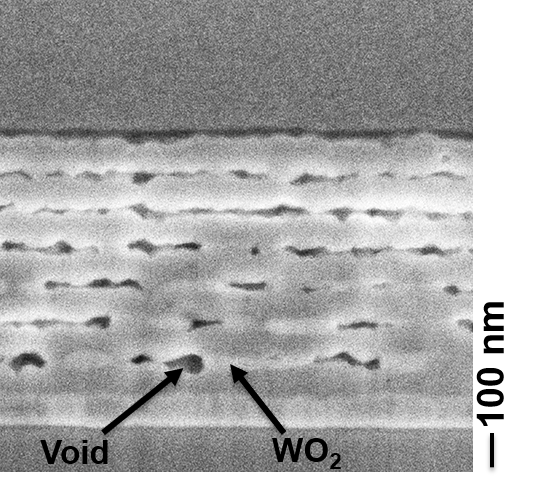


**Supplementary Figure 7 | Formation of voids and WO_2_**. SEM image shows the cross-sectional view of emitter structure after annealing at 1300 °C under 2×10^-3^ mbar vacuum pressure.

A typical SEM image of the emitter structure annealed at 1300 °C confirms the formation of WO_2_ (white regions in the W films) and voids due to the sublimation of WO_2.9_. By increasing the annealing temperature to 1500 °C, brown coloured substance, see Supplementary Figure 3c, is coated on the radiation shield of the heating chamber confirming the sublimation of WO_2_^1-4^.


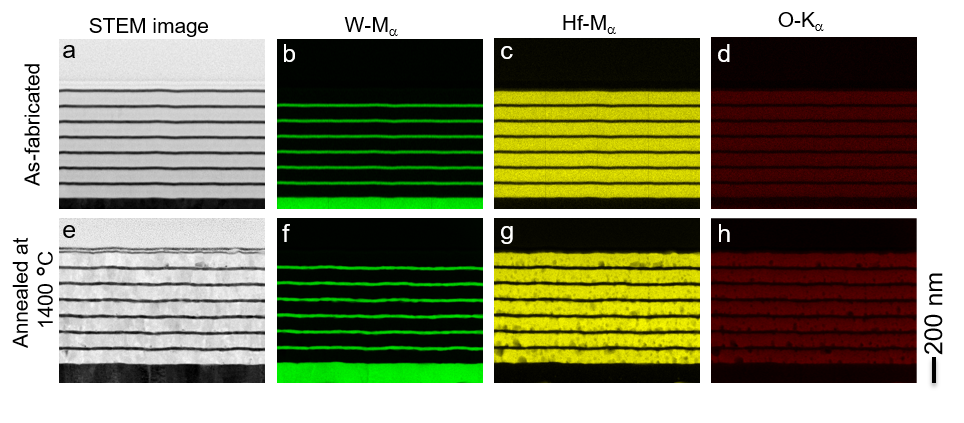


**Supplementary Figure 8 | Structural stability of the emitter structure at 1400 °C**. STEM and element mapping of W, Hf and O for as-fabricated and annealed for 6 hours at 1400 °C under 2×10^-6^ mbar vacuum pressure.


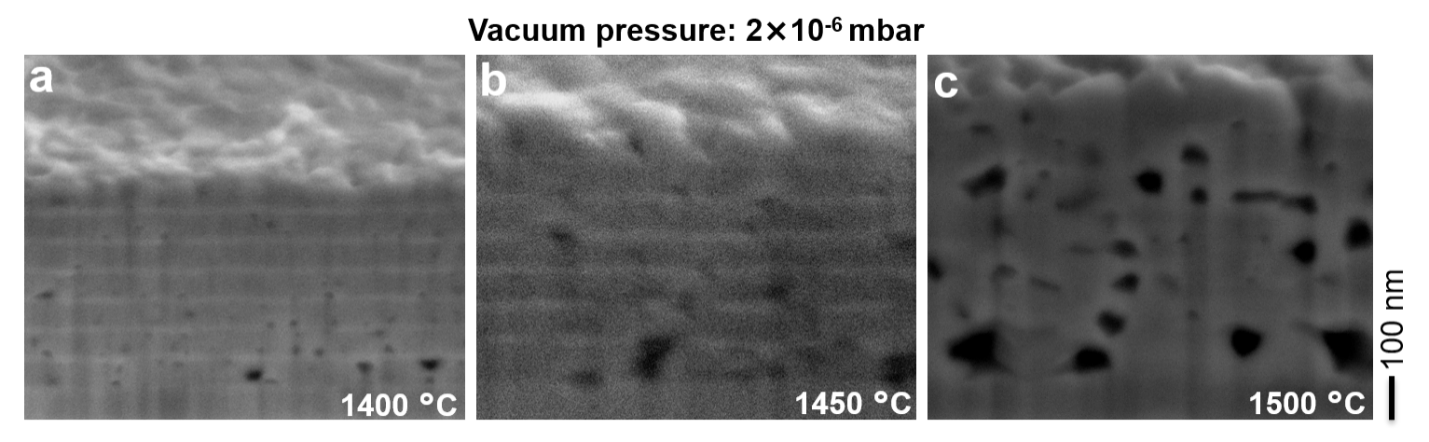


**Supplementary Figure 9 | Cross-section scanning electron micrographs of the emitter structure at high-temperatures and high vacuum conditions (2×10^-6^ mbar)**. Structural morphology of the emitter structure annealed for 6 h at 1400 (**a**), 1450 (**b**) and 1500 °C (**c**), respectively.


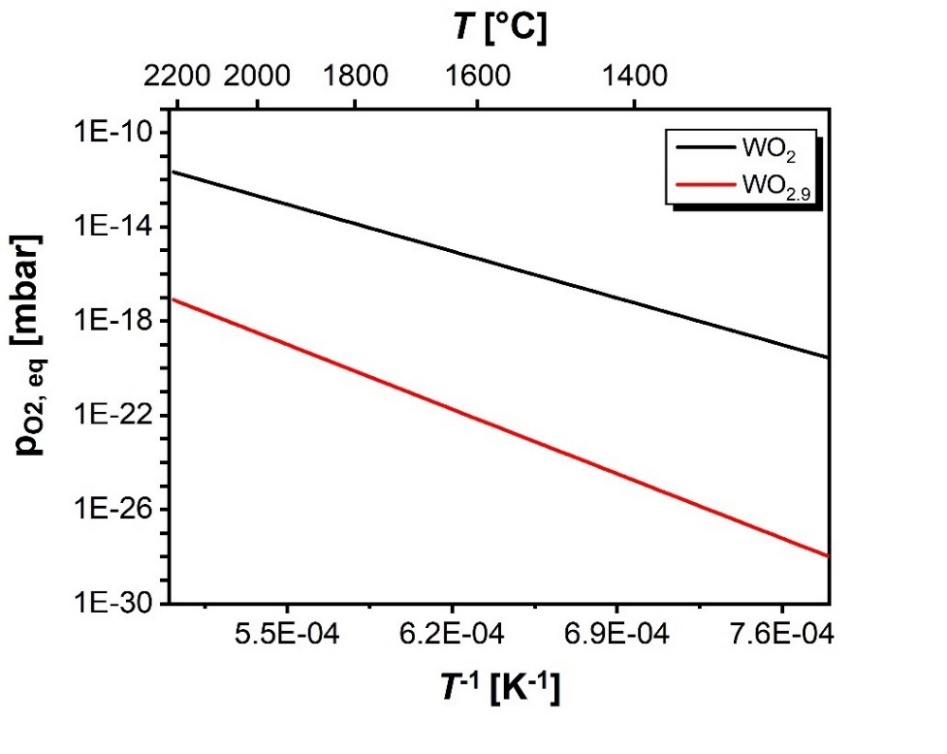


**Supplementary Figure 10 |** Oxygen partial pressure at equilibrium for the formation of tungsten oxides at 2×10^-2^ mbar, and different temperatures.

Oxygen partial pressure at equilibrium was calculated using the following equation:

$p_{O_{2,}eq}=\frac{RT}{K+\frac{RT}{p_{O_{2}}}},$ (Eq. S1)

$$\mathrm{where} K=\frac{-{\Delta G}^{0}}{RT}$$

Here, $p_{O_{2}}, p_{O_{2,}eq}, R, T,K, {\Delta G}^{0}$corresponding to oxygen partial pressure, oxygen partial pressure at equilibrium, universal gas constant, temperature, equilibrium constant and Gibbs free enthalpy of formation of the W oxides.

In order to exclude a redox reaction according to:

HfO_2_ + W 🡪 Hf + WO_2_ (Eq. S2)

At elevated temperatures we have to calculate the Gibbs free enthalpy $\Delta G_{WO_{2}}^{0}$ of the reaction consisting of the formation reaction of WO_2_:

W + O_2_ 🡪 WO_2_ (Eq. S3)

and the reverted reduction reaction of HfO_2_, i.e. its formation:

Hf + O_2_ 🡪 HfO_2_ (Eq. S4)

with $\Delta G_{\mathrm{Hf}O_{2}}^{0}$.

In the literature^5-8^, we find values of $\Delta G_{WO_{2}}^{0}=-530 kJ/mol$ and $\Delta G_{\mathrm{Hf}O_{2}}^{0}=-909 kJ/mol$, i.e. for the reduction of HfO_2_ one would conclude on $\Delta G_{\mathrm{Hf}}^{0}=909 kJ/mol$ at room temperature. Overall, at room temperature this gives a total free reaction enthalpy of $\Delta G=+379 kJ/mol$. For elevated temperatures $T$ this value scales according to Gibbs-Helmholtz equation:

$\Delta G=\Delta H-T\Delta S$ (Eq. S5)

with the reaction enthalpy $\Delta H$ and the reaction entropy $\Delta S$; the latter forming the $T$-sensitive term. Since both of these values were not available from the literature, we compare them with those for typical other transition metal oxides, e.g. FeO which has $\Delta H=-251 kJ/mol$and $\Delta S=61 J/mol/K$. Consequently, the $T$-dependent entropic term must equal $T=4114 K$ in order to invert to $\Delta G<0$ and reaction Eq S2 to occur. At 1400 °C (1673 K) this would mean that $\Delta S$ of reaction Eq S2 must be higher by a factor of roughly 2.5× compared with FeO formation which, when comparing typical values amongst transition metal oxides will hardly be the case. Consequently, we can still exclude redox reaction Eq S2 to occur even at 1400 °C.


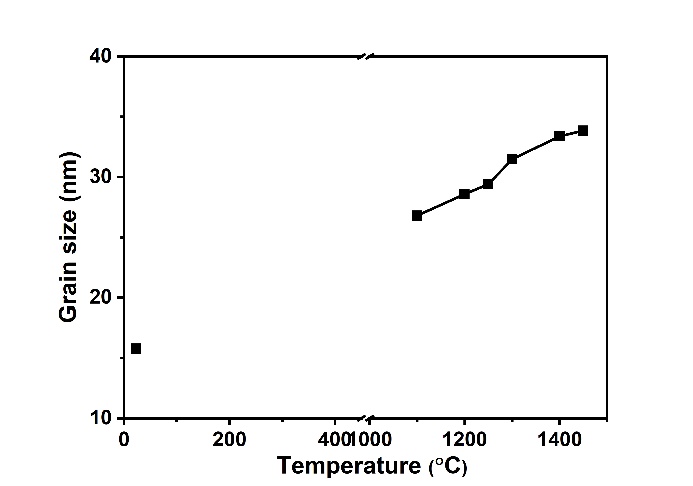


**Supplementary Figure 11 |** Grain size of monoclinic HfO_2_, calculated from the dominant (-111) peak, after annealing at different temperatures for 6 h under 3×10^-5^ mbar vacuum condition.

HfO_2_ grain size is calculated using Scherrer equation:^9,10^

$$Grain size=\frac{K\lambda}{\beta Cos\theta}$$

Where $K$, $\lambda, \beta$ and $\theta$ corresponding to dimensionless shape factor, X-ray wavelength (Cu K_a_=0.154055Å), FWHM of the fitted peak in radians and Bragg angle in degrees, respectively.

**References:**

1 Cifuentes, S. C., Monge, M. A. & Pérez, P. On the oxidation mechanism of pure tungsten in the temperature range 600–800°C. *Corrosion Science* **57**, 114-121 (2012).

2 Tilley, R. J. D. *Colour and the Optical Properties of Materials: An Exploration of the Relationship Between Light, the Optical Properties of Materials and Colour*. (John Wiley & Sons, Ltd, 2011).

3 Li, Y. H. *et al.* Local atomic structure modulations activate metal oxide as electrocatalyst for hydrogen evolution in acidic water. *Nature Communications* **6**, 8064 (2015).

4 Zhou, Z. *et al.* Tungsten Oxide Nanorods: An Efficient Nanoplatform for Tumor CT Imaging and Photothermal Therapy. *Scientific Reports* **4**, 3653 (2014).

5 Martienssen, W. & Warlimont, H. *Springer Handbook of Condensed Matter and Materials Data*. (Springer, 2005).

6 Barin, I. & Platzki, G. *Thermochemical Data of Pure Substances*. Vol. 304 (Wiley Online Library, 1989).

7 B. Reed, T. *Free energy of formation of binary compounds.* (MIT Press, 1971).

8 Wriedt, H. A. The O-W (oxygen-tungsten) system. *Bulletin of Alloy Phase Diagrams* **10**, 368-384 (1989).

9 Langford, J. I. & Wilson, A. J. C. Scherrer after sixty years: A survey and some new results in the determination of crystallite size. *Journal of Applied Crystallography* **11**, 102-113 (1978).

10 Jeffery, G. A. Elements of x-ray diffraction (Cullity, B. D.). *Journal of Chemical Education* **34**, A178 (1957).
